# The effectiveness of tobacco cessation programs for university students: A systematic review and meta-analysis

**DOI:** 10.18332/tid/162001

**Published:** 2023-06-02

**Authors:** Kanyaphat Setchoduk, Panan Pichayapinyo, Punyarat Lapvongwatana, Natkamol Chansatitporn

**Affiliations:** 1 Department of Public Health Nursing, Faculty of Public Health, Mahidol University, Bangkok, Thailand; 2 Department of Biostatistics, Faculty of Public Health, Mahidol University, Bangkok, Thailand

**Keywords:** systematic review, meta-analysis, tobacco cessation, university students

## Abstract

**INTRODUCTION:**

This systematic review and meta-analysis aimed to explore the existing tobacco interventions and synthesize whether those interventions affected tobacco use among university students.

**METHODS:**

We searched and found 1799 studies in PubMed, ClinicalKey for Nursing, Embase, and SCOPUS between 2009 and 2022. The risk of bias was assessed using similar criteria for RCT and non-randomized studies guided by the Cochrane Handbook for Systematic Reviews. The heterogeneity of studies was evaluated using Cochran’s Q and I^2^ index. The GRADE system was used to distinguish the quality of evidence, and Egger’s linear regression test was performed to assess publication bias.

**RESULTS:**

Eighteen studies used data extraction and analyses, and only eleven were meta-analyzed, which found that the estimate obtained via the fixed-effects model was statistically significant. Technology-based and motivational interview interventions found pooled ORs of statical significance, while reinforcer interventions showed the smallest effect size. The level of heterogeneity was considered substantial. The assessment for quality of evidence showed low overall certainty of evidence due to imprecision of outcome and suspicion of publication bias. Egger’s test showed no publication bias among included studies (p=0.38).

**CONCLUSIONS:**

There were numerous tobacco cessation interventions for university students, but the most effective intervention to change tobacco consumption behavior was still inconclusive and uncertain.

**TRIAL REGISTRATION:**

This systematic review was registered with PROSPERO. The registration number is CRD42019142491.

## INTRODUCTION

Tobacco use is one of the leading causes of chronic diseases and mortality worldwide. Despite governments implementing robust tobacco control policies, tobacco causes the premature death of more than 8 million of the world’s population yearly, especially in low- and middle-income populations^1^. Several strategies for tobacco cessation have been used. They include creating smoke-free environments, educational campaigns, quitlines, air quality policies, face-to-face sessions, mobile web-based applications, and blended strategies^2-4^. Outcomes varied from cognitive changes, such as knowledge, to behavior changes, such as refraining from smoking initiation or quitting smoking^5-7^. Most interventions have been designed to motivate and assist people in stopping tobacco use in adolescence and as adults in community settings, schools, and workplaces^5-8^. Consequently, a reduction in the overall prevalence of tobacco use has occurred in many countries.

Tobacco use among youth is now an alarming trend in some countries^9^. Specifically, in two-thirds of 31 countries where data are available, more than 30% of current smokers started smoking daily by the age of 16 years^10^ or among young people aged 15–24 years globally. The average rate of tobacco use worldwide was 17.0% in 2015 and has tended to increase every year^11^. Noticeably, the literature reviews regarding tobacco cessation programs specifically tailored and applicable to solve university or college students’ unique tobacco use issues are limited.

This systematic review and meta-analysis aimed to explore the existing type of tobacco interventions and synthesize whether those interventions had any effect on tobacco use among university students.

## METHODS

### Data sources and search strategies

This systematic review was registered with PROSPERO, the prospective international register of systematic reviews. The registration number was CRD42019142491 on 23 October 2019. Articles published from 2009–2022 were searched in PubMed, ClinicalKey for Nursing, Embase, and SCOPUS.

To be included in this review, a study followed the PICO key terms, and all words were based on medical subject headings (Mesh):

- Population (P): university students, college students, undergraduate student
- Intervention(s) (I): tobacco product use, smoking, cigarette smoking, waterpipe tobacco, electronic cigarettes, chewing tobacco, hookah tobacco, and dokha
- Comparator(s) (C): the group that received no intervention or control group.
- Outcomes (O): tobacco cessation, quit cigarette smoking

### Eligible studies

**Figure 1:**
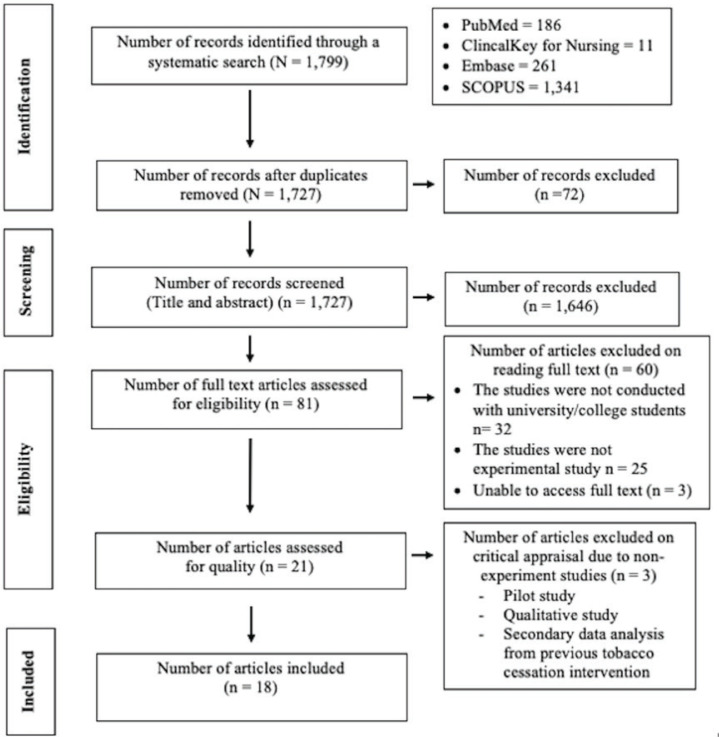
PRISMA flowchart of study selection

Articles searched were restricted to articles in English published as full text, including randomized controlled trials or quasi-experimental studies, with no age or gender limitation. Studies were excluded if they were reviews, case reports, or cross-sectional studies. The search results were reported in the final systematic review and meta-analysis and are presented in the Preferred Reporting Items for Systematic Reviews and Meta-analyses (PRISMA) flow diagram (Figure 1).

### Quality evaluation and data extraction

Following the search, all identified citations were collated and uploaded into bibliographic software, and duplicates were removed. Titles and abstracts were then screened by two independent reviewers (KS, PP) for abstract and full-text assessment against the inclusion criteria for the review. Any disagreements between the reviewers at each stage of the study selection process were resolved through discussion.

Data from included studies involved specific details about the setting, sample size, population characteristics, methodology, interventions, outcome measurements, and descriptions of main results.

### Statistical analysis and synthesis of results

Articles were pooled in a statistical meta-analysis using Review Manager version 5.4. Effect sizes were expressed as odds ratios (ORs) for dichotomous data, and their 95% confidence intervals (CIs) were calculated for analysis. Heterogeneity between the studies was defined as p<0.1 or I^2^ >50% using Cochran’s Q and I^2^ index, respectively. I^2^ heterogeneity was categorized as: low, <25%; moderate, 25–75%; and high, >75%. In the case of heterogeneity across the studies, a random-effect model was used to aggregate effect size across studies. If not, the fixed-effect model was employed. Forest plots were used to present the pooled estimates of odds ratios and the 95% confidence intervals.

The GRADE system was used to distinguish the quality of evidence from recommendations about the use of the intervention, based on five conditions, including study limitations, directness, consistency, precision, and report bias^12^. Quality ratings were made through the GRADE Pro program (https://gdt.gradepro.org). GRADE rates the quality of a body of evidence as high, moderate, low, or very low. Also, the publication bias was assessed by observing the symmetry of funnel plots and with Egger’s linear regression test, performed by the program STATA, version 15, with a p<0.05 as a statistically significant level.

## RESULTS

### Search results

A total of 1799 records were identified in the initial search stage. After duplicates were removed and titles and abstracts screened, 1646 records were excluded. Eighty-one full-text articles were retrieved and assessed for eligibility. Then, 60 articles were excluded after reading the full-text articles. Twenty-one articles were evaluated for data quality, and another three were excluded on critical appraisal due to methodology incongruity with the review. Finally, 18 articles were included in the systematic review and meta-analysis.

### Study characteristics

Participants were 8186 students, with subjects per intervention ranging from 29 to 2127 and ages ranging from 16 to 25 years (Table 1). There were eleven randomized controlled trials (RCT)^13-23^ and seven non-RCT^24-30^. All studies reported changes in quitting tobacco use of quit durations ranging from 21 days to 6 months. Intervention types were grouped into two categories: technology-based interventions, which consisted of nine studies^14-19,21,27,28^ and the face-to-face approach, which consisted of nine studies also^13,20,22-26,29,30^. Specifically, thirteen studies implemented a single intervention^14,15,17-20,22,24-28,30^ whereas five studies combined multiple interventions to enhance the effectiveness of tobacco cessation programs^13,16,21,23,29^. Study characteristics are presented in Table 1.

**Table 1:** Characteristics of included studies

Authors Year	Setting	Sample size	Participant characteristics	Methodology	Intervention	Outcomes measurement	Description of main results
Thomas et al.^13^ 2015	USA [17 colleges and universities across USA]	1217 Single contest n=306 Single PLUS n=295 Multiple contests n=309 Multiple PLUS n=306	Students who had smoked at least one cigarette per day on ≥10 days in the past month (hookah users).	Two-by-two factorial randomized clinical trial with group allocation to four treatment arms	A single Quit and Win contest, with and without motivation and problem-solving counselling (MAPS) to multiple, concurrent contests with and without counselling.	Self-reported abstinence from cigarettes and all tobacco products at 1, 4, and 6 months. Urine cotinine test at 6 months post enrolment.	Hookah users, when compared to non-users, had a 36% decrease in odds of self-reported 30-day abstinence at 4 months (OR=0.64; 95% CI: 0.45–0.93, p=0.02). 63% decrease in odds in biochemically verified continuous abstinence at 6 months (OR=0.37; CI: 0.14–0.99, p=0.05).
El-Awaisi et al.^24^ 2017	Qatar	50	Students from four different health disciplines in Qatar (medicine, pharmacy, pharmacy technician, and public health).	A pre-post intervention research design using the Readiness for Interprofessional Learning Scale (RIPLS)	A three-hour IPE (Incorporating interprofessional education) activity focused on smoking cessation including lecture, VDO on motivational interviewing, and case scenario discussion.	Students’ attitudes score toward IPE	Most of the students reported having a positive attitude toward IPE; the number of students having a positive attitude toward IPE increased after the IPE session. The overall median (IQR) score increased from 82 (16) before the session to 84 (15) after the session.
Prochaska et al.^14^ 2014	USA [7 mental health settings in the San Francisco Bay Area]	47	Adolescents and young adults between the aged 13–25 years who reported smoking at least one cigarette in the past month and at least 100 cigarettes in their lifetime.	Randomized controlled trial	Intervention participants received computerized motivational feedback at baseline, at 3 months, and 6 months, and were offered 12 weeks of cessation counselling and nicotine patches.	7-day point prevalence abstinence at 3, 6, and 12 months Biochemical confirmation at 3, 6, and 12 months Smoking reduction 24-h quit attempts	47% of the sample reduced their smoking 80% quit for 24 h 11%, 13%, and 17% confirmed 7-day point prevalence abstinence at follow up at 3, 6, and 12 months, respectively, with no differences by treatment condition (p>0.400).
Chulasai et al.^15^ 2022	Thailand [5 universities in Chiang Mai Province, northern Thailand]	273	Participants were smokers who had smoked minimally one cigarette within the previous 30 days and interested in smoking cessation in the next 30 days.	An open-label, parallel, 2-group, RCT with a follow-up at 12 weeks	Quit with US (Application on smartphones comprised five main pages: Suggested by US Talk with US Quit with US Let US Help Success of US	7-day point prevalence at the follow-up at 12 weeks Exhaled CO concentration level ≤6 ppm Change in smoking behaviors	Smoking abstinence rate was 58.4% (80/137) in intervention group and 30.9% (42/136) in control group (RR=1.89; 95% CI: 1.42–2.52, p<0.001). The mean daily cigarette consumption in intervention group was −4.50 (3.74) and −3.28 (3.50) in control group, p=0.010. The mean exhaled CO concentration level in the intervention group was −3.60 (3.56) and −2.44 (3.83) in control group, p=0.016.
Berg et al.^25^ 2022	USA [Students from 2 colleges in Massachusetts]	Pre-test n=418 Post-test n=640	Students who had smoked at least one cigarette within the past 30 days and had smoked >100 cigarettes in lifetime.	A pretest-posttest natural experiment between two college campuses in Massachusetts	One campus enacted a campus-wide smoking ban and acted as the experimental campus. The control campus was chosen based on its similarity in terms of its size, location, and shared liberal arts approach.	Beliefs about smoking Behavioral norms Attitudes toward a campus-wide smoking ban	There were no significant differences of students who reported smoking at least one cigarette in the past 30days between the two school before or after the ban. There was not a statistically significant decrease in percentage of students who had smoked ≥100 cigarettes in their lifetime, from 2014 to 2018. Attitudes toward smoking, perceived smoking, and attitudes toward a campuswide smoking ban did improve significantly.
Prokhorov et al.^16^ 2021	USA [3 campuses at the Houston Community College]	636 GSE n=81 GCE n=79 GSR n=73 GCR n=82 LSE n=77 LCE n=77 LSR n=81 LCR n=86	Young adults aged 18–25 years, enrolled in a community college.	A 6-month randomized trial with 8 arms based on a combination of 3 message categories: framing, depth, and appeal.	Participants received SMS text messages on their mobile phones for free in 2 waves or campaigns. Each campaign comprised 2 SMS text messages per day for 30 days (120 messages).	Self-reported attention level to the messages perceived CTP (conventional tobacco products) risk and perceived NETP (new and emerging tobacco products) risk.	Participants had a significant increase in perceived NETP risk over time (p<0.001); however, participants had a marginal increase in perceived CTP risk (p=0.008). A significant increase in perceived NETP risk among participants who received rational messages (p=0.005), emotional messages (p=0.006), simple messages (p=0.003), and gain-framed messages (p=0.003).
Joveini et al.^26^ 2020	Iran	150	Male undergraduate students, who were regular hookah smokers (at least once a month).	Quasi-intervention study	The education program was divided into two stages: 1) Motivation and volition. 2) The intervention group received seven sessions of education during these two stages, while the control group did not receive any education.	Intention to quit hookah Abstinence from tobacco use.	44.1% of intenders in the intervention group and 9.4% in the control group quitted hookah after 6 months of the intervention. 19 out of 71 students in intervention group and 6 out of 67 in control group successfully quitted hookah in 12 months after the intervention.
Romero-López at al.^27^ 2020	Spain	29	Nursing and Physiotherapy students who were regular smokers.	A two-phase pilot study was conducted: The first phase was cross-sectional, and the second phase was a before-and-after intervention.	An intervention based on the use of information technologies in the motivation to quit smoking.	Means of the Richmond questionnaire Dependence on nicotine through the Fagerström questionnaire	3.45% of the participants had a high level of dependence; and 6.90%, a high level of motivation. The level of motivation did not change after the intervention (p=0.10).
Scholten et al.^17^ 2019	Netherlands	144	Age 16–26 years, at least a weekly smoker, motivated to quit smoking for at least four weeks during study participation.	Two-armed randomized controlled trial (RCT)	A peer-based social mobile game intervention: participants in the game group were instructed to play the game at least once per day for 2 to 5 minutes, and they received tailored prompts to keep them engaged with the intervention and remind them of the purpose of the game.	Weekly smoking behaviour Abstinence Intervention dose Text-based analyses Peer- and engagement-related factors	Similar reductions in weekly smoking levels and similar abstinence rates for both groups. The longer participants played HitnRun, the lower their weekly smoking levels were. A chi-squared test revealed no significant effect for group on abstinence levels at post-test, and follow-up. Abstinence rate (post-test) Intervention group 25/47 Control group 25/47 Abstinence rate (follow-up) Intervention group 21/51 Control group 22/50
Haug et al.^18^ 2017	Switzerland [24 vocational schools, incorporating 360 classes in total]	2127 MCT n=741 MCT+ n=730	Students who smoked tobacco regularly (≥4 cigarettes over the preceding month and at ≥1cigarette within the preceding week) and owned a mobile phone.	Two-arm, parallel-group, cluster-randomized controlled trial	Web- and text messaging-based program using the MobileCoach system providing individually tailored mobile phone text messages to support smoking cessation for a 3-month period.	7-day point prevalence of smoking abstinence Stage of change Quit attempt Quantity of alcohol consumption	7-day point prevalence rates for smoking abstinence and the pre-to-post intervention differences in the number of cigarettes smoked daily for both study groups. 7-day smoking abstinence rate at follow-up was 13.9% (77/552) in the MCT group and 15.0% (84/559) in the MCT+ group (CC: p=0.61; ITT: p=0.82). The mean number of cigarettes smoked per day decreased from baseline to follow-up by 2.8 (7.6 (7.1)) in MCT and 2.7 (7.1 (6.6)) in MCT+ group (CC: p=0.97; ITT: p=0.93).
Jorayeva et al.^28^ 2017	USA [Metropolitan university in the Mid- South region of the USA]	33	Age 18–24 years, current smoking status, active university enrolment, ability to read and understand English, ability to send and receive text messages, and access to the Internet.	A quasi-experimental one-group pretest-posttest design with repeated measures	Intervention text messages were built on the fundamental processes of motivational interviewing by engaging the smoker in procedure: a respectful relationship, focusing on the goals, evoking change talk and developing a change plan.	Severity of nicotine addiction Number of cigarettes per day Psychological needs satisfaction Autonomous motivation Smoking cessation self-efficacy Readiness to quit	Students’ level of autonomy and relatedness needs satisfaction, autonomous motivation, and smoking cessation selfefficacy increased (p<0.05). Rate of daily smoking declined (p<0.05) over time. Competence need satisfaction, readiness to quit smoking and severity of nicotine addiction remained unchanged. Smoking cessation self-efficacy was the strongest predictor of smoking behavior change in college students.
Müssener et al.^19^ 2016	Sweden [25 student healthcare centers at all universities and colleges in Sweden]	1590	Students who were daily or weekly smokers and were willing to set a quit date for smoking cessation within the 4 weeks following enrolment.	A single-blind, 2-arm, randomized clinical trial of an SMS text-based messaging smoking cessation intervention in which participants were randomized to an immediate-intervention.	SMS text-based messaging smoking cessation intervention: The Nicotine Exit (NEXit) consists of 157 text messages, with the option to request extra messages when having cravings to smoke, relapse, or concerns about weight gain. The participants received 4 to 5 text messages per day in the first week, followed by a decreasing number of messages throughout the 12-week intervention.	Self-reported prolonged abstinence 4-week point prevalence of not having smoked a single cigarette at the time follow-up Self-reported, 7-day point prevalence of smoking abstinence The mean number of quit attempts The number of uses of other smoking cessation services The number of cigarettes smoked weekly at the time of follow-up	Eight-week prolonged abstinence was reported by 203 participants (25.9%) in the intervention group and 105 (14.6%) in the control group. 4-week point prevalence of complete cessation was reported by 161 (20.6%) and 102 (14.2%) participants, respectively. A mean (SD) of 3.9 (0.37) months after the quit date. The adjusted odds ratios (95% CIs) for these findings were 2.05 (1.57–2.67) and 1.56 (1.19–2.05), respectively.
Pardavila-Belio et al.^20^ 2015	Spain [University of Navarra, in two close urban capital cities]	255	Undergraduate or Master’s students, aged 18–24 years, who had smoked an average of ≥1 cigarette a week within the last 6 months.	Pragmatic randomized controlled trial Intervention was based on the Theory of Triadic Influence (TTI)	A multi-component intervention including motivational interview and online self-help material to change student perception of tobacco and increase self-efficacy.	Proportion of students who stopped smoking. Self-report,7-day abstinence from smoking at 6 months Urine cotinine analysis Mean of smoked cigarettes Quit attempts Stages of change	At the follow-up at 6 months, the smoking cessation incidence was 21.1% (30/133) in the intervention group and 6.6% (10/122) in the control group (difference=14.5, 95% CI: 6.1–22.8; relative risk=3.41; 95% CI: 1.62–7.20). The mean number of cigarettes at 6 months was significantly different (difference=-2.2, 95% CI: -3.6 – -0.9).
Schoonheim-Klein et al.^29^ 2013	Netherlands	255 Control n=70 MI-1 n=58 MI-2 n=77 MI-3 n=50	Participant were both patients and dental students: Dental students n=402 patients, including 109 smokers (27%), received periodontal therapy in the four consecutive courses	Pre–post study design	The intervention enhances the capabilities of the students to apply MI counseling for smoking cessation to dental patients.	Smoking habits Attitudes and knowledge related to tobacco cessation Perceived quality of the education in MI of the students	Five out of 13, reflected a quit rate of 38% in MI-3 The number of cigarettes smoked by the students decreased significantly from 11 to 7 cigarettes per day in MI-3. Smoking status after intervention Control = 10/70 MI-1 = 12/58 MI-2 = 8/77 MI-3 = 13/50
Peng et al.^21^ 2013	Taiwan	116	Students who were found smoking on campus by a Military Officer in Taiwan universities.	A randomized controlled research design Three groups received different intensity of assessment and intervention schedules over a 9-week period.	The web-phone intervention (WPI) based on the TTM theoretical, Motivational Interviewing, Cognitive Behavioral Therapy was used. The WPI delivered phone calls that assessed participants’ smoking status and based on their responses, delivered motivational and educational recorded messages.	Stage of change (SOC) Self-efficacy (SE) Decisional balance	After 4 weeks the participants in both the experimental group and comparison group improved on self-efficacy (SE) and stage of change (SOC) toward smoking cessation. After another 5 weeks, their SE remained significantly improved, but SOC did not.
Harris et al.^22^ 2010	USA [13 sororities and 17 fraternities in one large Midwestern university]	452	Students who reported smoking cigarettes one or more of the past 30 days, had not used medications to help quit smoking in the past 30 days, and were at least 18 years old.	A group randomized controlled trial	In the treatment condition, participants received MI focused on motivating and assisting participants to quit cigarette smoking while in the comparison condition participants received MI focused on increasing consumption of fruits and vegetables to at least 5 servings a day.	Timeline Follow-Back Method at all time points Saliva samples for cotinine Quit attempt for at least 24 h Motivation and confidence to quit Number of five best friends who smoke Romantic partners’ smoking status Self-identification as a smoker Days consuming at least one drink of alcohol Servings of fruits and vegetables eaten per day Dependence using the 10-item Hooked on Nicotine Checklist	No significant differences were found for 30-day cessation between treatment and comparison at end of treatment (31.4% vs 28%, OR=1.20; 95% CI: 0.72–1.99) or at follow-up (20.4% vs 24.6%, OR=0.78; 95% CI: 0.50–1.22). Predictors of cessation at follow-up, regardless of condition, included more sessions attended (OR=1.2; 95% CI: 1.1–1.8) and more cigarettes smoked in 30 days at baseline (OR=4.7; 95% CI: 2.5–8.9). The odds of making at least one quit attempt were significantly greater for those in the smoking group at end of treatment (OR=1.75; 95% CI: 1.11–2.74) and follow-up (OR=1.66; 95% CI: 1.11–2.47). 30-day quit rates for end of treatment (EOT) and follow-up (FU) for treatment groups: EOT (Smoking) 77/245 (F&V: Control) 58/207 FU (Smoking) 50/245 (F&V: Control) 51/207
Wang et al.^30^ 2010	Taiwan [Three universities in Taiwan]	62	Students who smoked and had levels of exhaled CO >6 ppm and serum cotinine levels >100 ng/mL	A quasi-experimental group design	Group 1 received the 10-week program with auricular acupressure plus multimedia instruction. Group 2 received auricular acupressure alone.	Level of exhaled CO, and serum cotinine level Self-efficacy Nicotine dependence The smoking cessation self-efficacy	Statistical between-group differences existed in psychological factors of smoking cessation self-efficacy and nicotine dependence, but not in physical factors of carbon monoxide and cotinine. Rate of cessation (CO <6 ppm) group 1 = 12/30 group 2 = 11/32
Tevyaw et al.^23^ 2009	USA [Colleges and universities in a north-eastern U.S. state]	110 Group 1: CM + MET (n=28) Group 2: CM + REL (n=27) Group 3: NR + MET (n=27) Group 4: NR + REL (n=28)	Students who were daily smokers, aged 18–24 years and have a breath CO level of at least 10 parts per million (ppm) at screening.	A randomized Clinical trial: A 2×2 design (psychosocial condition × reinforcement condition)	The psychosocial condition compared three individual sessions of Motivational Enhancement Therapy (MET) to three individual sessions of progressive muscle relaxation control (REL) treatment. The reinforcement condition compared 3 weeks of Contingency Management (CM) to 3 weeks of Noncontingent reinforcement (NR).	Expired CO Saliva samples for cotinine A 30-day timeline follow back (TLFB) interview Past 30-day use of other forms of tobacco Contemplation ladder Modified Fagerstrom Tolerance Questionnaire Attendance at the three intervention sessions and the 42 CO readings Treatment adherence, satisfaction, and interest in quitting smoking	CM resulted in significantly lower CO levels and greater total and consecutive abstinence during the intervention. Point prevalence abstinence: there were no significant differences between groups. Of those in CM, 6.1% (3/49) were abstinent, versus 0% (0/43) in NR, χ^2^(1, N=92)=2.72, ns, h=0.50. Abstinence rates in MET (2.0%) and REL (4.8%) were comparable, χ^2^ (1, N=92)=0.55, ns, h=0.16. Point prevalence abstinence. Rates of verified abstinence at each follow-up were low: 6.4% (7/109) were confirmed abstinent at 1 month, 4.8% (5/105) at 3 months, and 3.8% (4/104) at 6 months.

### Types of intervention

*Technology-based approach*

Half of the included studies (9 out of 18 studies) selected technology-based smoking cessation interventions, comprising four mobile text messaging^16,18,19,28^, a web platform^27^, an automated web-phone intervention^21^, a smartphone application for smoking cessation^15^, a peer-based social, mobile game intervention^17^, and a computerized motivational feedback program^14^. The details of each technology-based smoking cessation intervention are described below.

The elements of mobile text messages differ across studies. Features included making a public declaration about quitting (i.e. telling friends about the quit attempt), asking friends and relatives for support, using problem-solving tips and distraction techniques, and the option to text for more help if craving to smoke or smoking^19^. In addition, there was an option to request extra messages in some interventions if subjects had cravings to smoke, relapsing, or concerns about weight gain^16,18,19^.

The smartphone application named ‘Quit with US’ consisted of five main pages designed to help university/college students quit smoking, including: 1) offering information regarding the disadvantages of tobacco smoking and recommending to quit (Suggested by US); 2) arranging the follow-up communication with experienced pharmacists (Talk with US); 3) assessing and assisting quitting smoking with a personalized quit plan (Quit with US); 4) helping to quit smoking by suggesting coping methods for nicotine craving and unintentional smoking (Let US help); and 5) arranging a self-monitoring of quitting smoking (Success of US)^15^.

An automated web-phone intervention system was used as a motivational/educational message delivered by phone calls. It aimed to assess participants’ smoking status and provide motivational and educational recorded messages based on their responses^21^.

A peer-based social, mobile game intervention called HitnRun was designed to be played during individualized moments of high craving, stress, or boredom. The peer interaction was a game-based experience to support and reinforce desired smoking behavior. The participants in the game group were encouraged to play the game at least once per day, and tailored prompts were sent to keep them engaged with the intervention and remind them of the purpose of the game^17^.

A web platform-based intervention uploaded the information, images, and videos regarding the health risks and life experiences of ex-smokers. In addition, the web page provided a space where people could show their experience, recommend their ideas, and answer something in the open discussion forum^27^. Also, a computerized motivational feedback program used a two-staged treatment approach that combined a motivational engagement program and cognitive-behavior treatment (CBT)-oriented individual cessation counseling^14^.

*Face-to-face approach*

Nine studies implemented this approach in various interventions, including a reinforcer intervention such as contingency management^23^, a Quit & Win contest^13^, three motivational interviews^20,22,29^, four health education-based programs^24-26,29^, and an acupressure intervention^30^ as presented below.

Contingency management (CM) principally provides reinforcers contingent on abstinence or reduction of substance use as a set target. The critical components of CM include: 1) obtaining objective evidence of abstinence or another target behavior; 2) provisions of reinforcers, such as money or vouchers, when the target behavior is accomplished; and 3) withholding reinforcers when the target behavior does not occur^23^. The Quit & Win contest focused on abstinence from all tobacco products^13^.

Motivational interview (MI) was the single approach in three studies^20,22,29^ and combined with other interventions to sustain the desired outcome of cessation^23^. One study implemented the session of MI that included a face-to-face 50-minute meeting that mainly reinforced the decision to change, elaborating a personal plan to stop smoking^20^. Another study conducted four one-on-one sessions of MI with a trained counsellor, and the contents were focused on motivation and assisting participants in quitting cigarette smoking^22^.

Health education-based programs were implemented at both individual^26,29^ and campus/institutional levels^24,25^. One study had two stages of a seven-session education. The first stage comprised three education sessions to develop an intention to quit tobacco use. The second stage included four education sessions to promote coping, recovery, coping, and action planning for smokers who wanted to quit^26^. One study implemented health education to impress students about tobacco-related health effects and tobacco cessation^29^. The other study used inter-professional learning among medicine, pharmacy, pharmacy technician, and public health students. Teaching activities included a short didactic lecture, videos on motivational interviewing, case scenarios, and group discussion^24^. Another study implemented a campus-wide smoking ban policy to create a campus-wide smoking ban and attitude change among college students^25^. One study used an auricular acupressure technique with multimedia instruction guided by expert physicians who practice traditional Chinese medicine^30^.

### Outcome measures

The primary outcomes of tobacco cessation programs across the included studies were diverse. The primary outcome was self-reported abstinence commonly measured in 7-day point-prevalence^14,15,18-20^. Some studies evaluated the point prevalence of not having smoked a single cigarette or self-report of continuous abstinence widely measured over thirty days^13,18,19^. One study measured the most prolonged duration of constant abstinence in the past 12 months^26^. In contrast, one study measured the shortage of abstinence in only the last 24 hours^17^. Most of the included studies verified and confirmed tobacco use abstinence by biochemically verifying self-reported tobacco abstinence by detecting cotinine in urine^13,20^ or saliva samples^14,21-23^. Another way of abstinence verification and confirmation use was expired carbon monoxide (CO) after completing intervention or follow-up periods^14,15,23,30^.

### Risk of bias within studies

The risk of bias was assessed using similar criteria for RCT and non-randomized studies guided by the Cochrane Handbook for Systematic Reviews of intervention^31^. One RCT study^23^ and three studies of non-RCT^26,29,30^ had some limitations in systematically randomizing the participants into intervention and control groups. In allocation concealment criteria, there were two non-RCT studies^29,30^ and one RCT study^23^ that did not conceal the allocation of the participants into intervention and control groups. Most of the included studies^13,15,17,19,20,22,23,26,29,30^ needed to provide more information about the blinding of outcome assessment for authors to make a judgment regarding detection biases.

**Figure 2:**
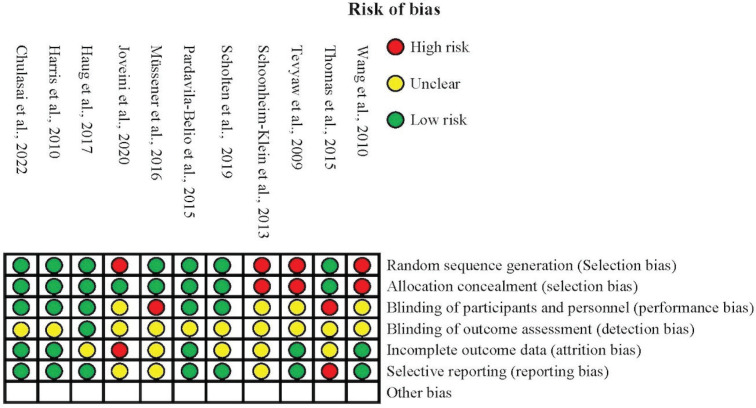
Risk of bias assessment

For attrition bias, some studies^13,17-19,29^ showed unclear completion of outcome data, whereas one study^26^ did not mention the completion of outcome data. Lastly, there were only three studies^19,26,29^ exhibiting unclear reporting bias, and one study^13^ showed a high risk of reporting bias due to the study selecting data from another study to analyze and report only the perspective of hookah use (Figure 2).

### Meta-analysis

**Figure 3:**
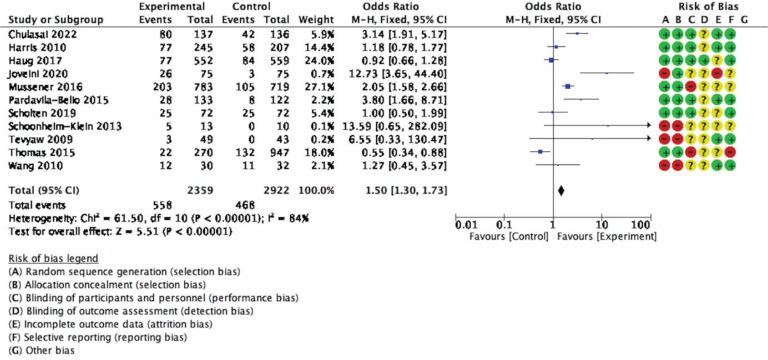
Forest plot for meta-analysis

The forest plot for meta-analysis provides information to assess statistical heterogeneity, as shown in Figure 3. Eleven studies were included to compare the types of intervention effectiveness with non-intervention. The effects on tobacco cessation across the included studies are generally consistent and favor intervention over the non-intervention group, showing a statistical difference. The analysis detected high heterogeneity (I^2^=84%, p<0.001), and the estimate obtained via the fixed-effects model was statistically significant (OR=1.50; 95% CI: 1.30–1.73, p<0.001).

**Figure 4:**
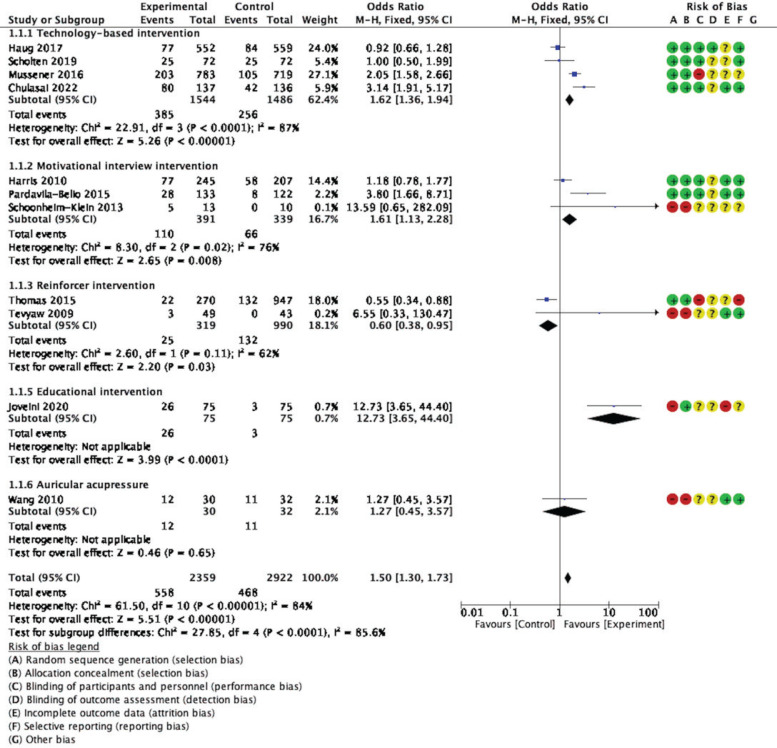
Forest plot for subgroup analysis (types of intervention)

Subgroup analysis based on types of intervention found that the technology-based^15,17-19^ and motivational interview interventions^20,22,29^ had equally pooled ORs with high heterogeneity (I^2^=87%, OR=1.62; 95% CI: 1.36–1.94, p<0.001; I^2^=76%, OR=1.61; 95% CI: 1.13– 2.28, p<0.01, respectively). Reinforcer interventions, including the Quit & Win contest^1^ and Contingency management^23^, found the smallest effect size with statically significant pooled ORs and moderate heterogeneity (I^2^=62%, OR=0.06; 95% CI: 0.38–0.95, p=0.03) (Figure 4).

**Figure 5:**
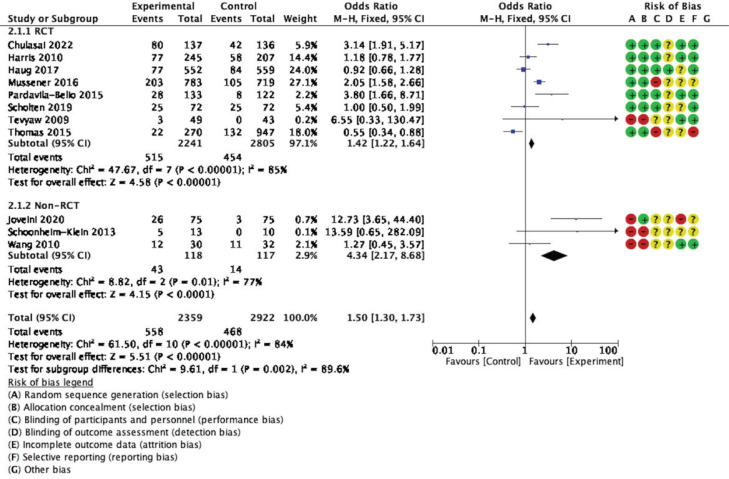
Forest plot of subgroup analysis (RCT vs non-RCT)

As shown in Figure 5, when evaluating the effects of interventions by a group of RCT versus non-RCT, the pooled OR of eight RCTs^13,17-20,22,23^ was less than three non-RCTs^26,29,30^ (I^2^=85%, OR=1.42, 95% CI: 1.22–1.64, p<0.001; I^2^=77%, OR=4.34; 95% CI: 2.17–8.68, p<0.001, respectively).

### Risk of bias across studies

**Figure 6:**
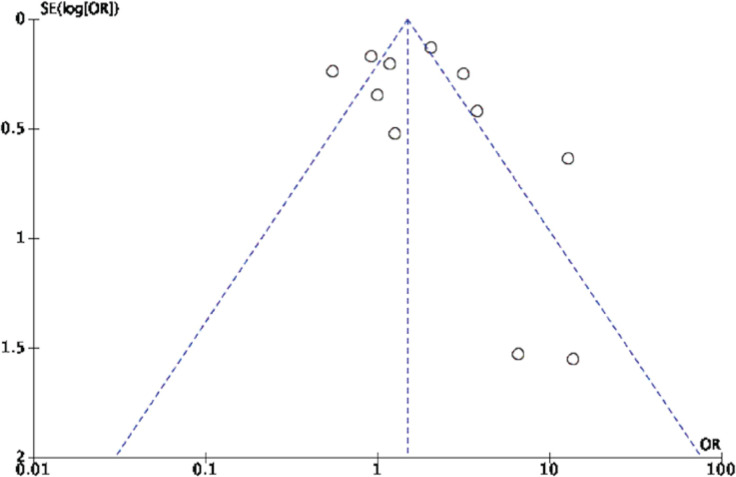
Funnel plots

The visual evaluation of publication bias revealed the asymmetrical distribution of the funnel plots. Most studies had larger sample and effect sizes, which might indicate publication bias in this review (Figure 6). The funnel plot analysis of types of intervention shows an asymmetrical distribution in the reinforcer interventions and the motivational interview interventions. The Egger’s regression test indicates no statistically significant publication bias among included studies (p=0.38). The assessment for quality of evidence showed low overall certainty of evidence due to the imprecision of outcome and suspicion of publication bias (Supplemenary file).

## DISCUSSION

This systematic review and meta-analysis investigated the effects of interventions on tobacco cessation among university students. Eighteen studies met the inclusion criteria and their data extracted and analyzed, and 11 studies were eligible for meta-analysis. The findings indicated that the pooled effect sizes for RCTs were significant but small for tobacco cessation compared to the non-RCTs. Among the studies reviewed, technology-based and motivational interview interventions greatly affected tobacco cessation.

High heterogeneity in effect size was found when types of intervention were compared to non-intervention. This heterogenicity may have been caused by an artefactual variation such as improper randomization and differential follow-up. In addition, another cause of heterogeneity in systematic reviews is actual variation in the treatment effect, including intervention factors such as dose, timing, or duration of treatment, and timing and event type of outcome^32^. Based on the risk of bias assessment of this review, improper random sequence generation, improper allocation concealment, lack of blinding of outcome assessment, and incomplete outcome data were observed. Thus, developing an effective intervention that is specific to university students is challenging.

Although asymmetry of the funnel plots was found in the reinforcer and the motivational interview interventions, there are many reasons for asymmetry. First, the quality of the trial design affected the apparent result. For instance, improper allocation concealment is associated with odds ratios exaggerated by 41%, whereas lack of blinding of outcome assessment is associated with odds ratios exaggerated by 17%^33^.

### Strengths and limitations

This study has several strengths. First, only RCT studies were included in this meta-analysis. Second, the number of participants was 8186 students, which allowed us to perform further analyses. Finally, this study assessed the risk of bias and the quality of evidence. However, this review is limited by the number of studies for meta-analysis due to differences in outcome measurement. Outcome measurements of tobacco cessation were also varied.

## CONCLUSIONS

The overall trend in tobacco consumption among young people has increased remarkably in the past decade. University students are a significant group of the young population defined by setting, demonstrating a high tobacco consumption rate. An effective tobacco cessation intervention for them should be identified, and it is essential. The findings from this review revealed that there were numerous tobacco cessation interventions for university students. However, an effective intervention to change tobacco consumption behavior within this population is still inconclusive.

## Data Availability

The data supporting this research can be found in the Supplementary file.
