# Supplementary material for: The effectiveness of tobacco cessation programs for university students: A systematic review and meta-analysis
Source: Tob Induc Dis. 2023 Jun 2;21:73. doi: 10.18332/tid/162001 (PMC10236936; doi:10.18332/tid/162001)
Supplement: Supplementary file 1 [file TID-21-73-s1.pdf]

Supplemantary file

Table 1. GRADE evident profile (for tobacco cessation rate outcome)

**Quality assessment**							**Participants**		**Magnitude of effect**		**GRADE for strength of evidence**
**Intervention**	**Methodology**	**Limitations**	**Inconsistency**	**Indirectness**	**Imprecision**	**Publication bias**	Control	Experiment	OR	95% CI	
Technology-based	4 RCTs	Serious	Not serious	Not	Not serious	Detected	1486	1544	1.62	1.36–1.94	⊕ ⊕ ⊕ ◯ Moderate
Motivational interview	2 RCTs<br>1 non-RCT	Not serious	Not serious	Not serious	Imprecision	Detected	339	391	1.61	1.13–2.28	⊕ ⊕ ◯ ◯ Moderate
Reinforcer	2 RCTs	Serious	Serious	Not serious	Imprecision	Detected	990	391	0.60	0.38–0.95	⊕ ◯ ◯ ◯ Very low
Other	2 non-RCTs	Serious	Serious	Not serious	Imprecision	Detected	107	105	3.96	1.94–8.11	⊕ ◯ ◯ ◯ Low

© 2023 Setchoduk K. et al.
